# Severe Japanese encephalitis with multiple intracranial hemorrhages: A case report

**DOI:** 10.1097/MD.0000000000017453

**Published:** 2019-10-11

**Authors:** Qiaochan Feng, Qi Chen, Xizhuang Bi, Shaoxia Yu, Jiahui Wang, Xuwen Sun, Chao Ren, Hong Liu, Lina Guan

**Affiliations:** a Department of Neurology; b Central Laboratory; c Neurosurgical Intensive Care Unit, Affiliated Yantai Yuhuangding Hospital of Qingdao University, Yantai, China.

**Keywords:** antiplatelet aggregation, cerebral amyloid angiopathy, cerebral infarction, Japanese encephalitis, vascular endothelial injury

## Abstract

**Rationale:**

Intracranial hemorrhage occurs infrequently in Japanese encephalitis (JE), and even less frequently with hemorrhage occurring twice. In this report, we describe the clinical features and outcomes of a patient with confirmed JE combined with hemorrhage twice.

**Patient concerns:**

The patient, a 71-year-old Asian woman, was admitted to the hospital with symptoms of hemiplegia following fever and diarrhea. Soon her condition worsened and a decreased level of consciousness, respiratory failure, and paralysis of extremities occurred.

The brain diffusion-weighted imaging sequence showed suspicious abnormal signals in bilateral thalami. Japanese encephalitis virus immunoglobulin M antibody was detected in her serum and cerebrospinal fluid samples, so the patient was diagnosed with JE. During treatment, her condition became aggravated and the brain computed tomography (CT) scan showed multiple lobar hemorrhages. One month later, the multiple lobar hemorrhages occurred again, as observed by a brain CT scan.

**Diagnosis:**

JE with multiple intracranial hemorrhages.

**Interventions:**

The patient was treated comprehensively, including surgery, lowering her intracranial pressure and ventilator-assisted breathing.

**Outcomes:**

One month later, the patient underwent another surgical procedure for intracranial hemorrhage and suffered a serious neurological disorder.

**Lessons:**

Severe intracranial hemorrhage may occur in elderly patients with JE, especially in those with poor vascular condition. Therefore, when treating such patients, great caution, as well as early detection and prevention, should be taken in case of the occurrence of severe intracranial hemorrhage.

## 1 Introduction

Japanese encephalitis (JE) is an acute infectious disease caused by the Japanese encephalitis virus (JEV), which is transmitted by hemophagous insects, such as mosquitoes. It is prevalent in summer in the tropical, subtropical, and temperate regions of eastern Asia, with a mortality rate of 20% to 40%.^[1]^ JE has many different manifestations, but it is commonly characterized by sudden fever, headache, vomiting, cognitive impairment, and disturbance of consciousness, which is sometimes accompanied by epilepsy, Parkinson's disease, and other symptoms; in severe cases, respiratory failure can also occur.^[2]^ There are few reports available on JE combined with cerebral hemorrhage, among which many described JE combined with bilateral thalamus or basal ganglia hemorrhage, while JE combined with multiple lobar hemorrhages has never been reported. Here we report such a case we encountered recently, together with literature review.

## 2 Case report

### 2.1 Patient concerns

The patient was an unemployed 71-year-old woman. She experienced fever on September 25, 2017 after eating a cold dish. Her highest body temperature was 40°C, with perichilum pain, nausea, vomiting, diarrhea, and defecation of unformed dilute stool. She visited the community hospital 2 days after the onset, and no abnormality was found in routine blood examination or C-reactive protein level (CRP). The patient received symptomatic treatment; however, during transfusion, her left hand could not hold things steadily while her left leg could still walk. She immediately received brain computed tomography (CT), which suggested senile brain changes. She was then transferred to our hospital 7 hours after feeling left limb weakness and was admitted for suspected cerebral infarction and infectious fever caused by acute gastroenteritis.

The patient had a previous history of surgical treatment for a fracture of the left femur in 2013. She was generally healthy and had no specialty in personal, menstrual, obstetrical, or family history, and did not smoke or drink.

### 2.2 Diagnosis and treatment

At the time of admission, the patient had a body temperature of 39.6°C, and she was conscious, mentally normal, and without aphasia. Her neck was soft, and Kernig's sign was negative. Her left nasolabial was shallow, and her tongue was toward the left when sticking out. Her left limb muscle strength was grade 5−, right limb muscle strength was grade 5, and bilateral pathological signs were negative. There was no abnormality in cardiopulmonary examination, and her abdomen was soft with no tenderness. The patient's blood gas analysis and electrolyte measurements were normal. Routine blood tests showed an elevated white blood cell count of 14.06 × 10^9^/L (normal range is 3.5–9.5 × 10^9^/L), a percentage of neutrophils of 85.4%, an elevated procalcitonin of 0.137 ng/mL (normal level is <0.05 ng/mL) and a CRP of 10 mg/mL (normal range 1–10 mg/mL).

**Figure 1:**
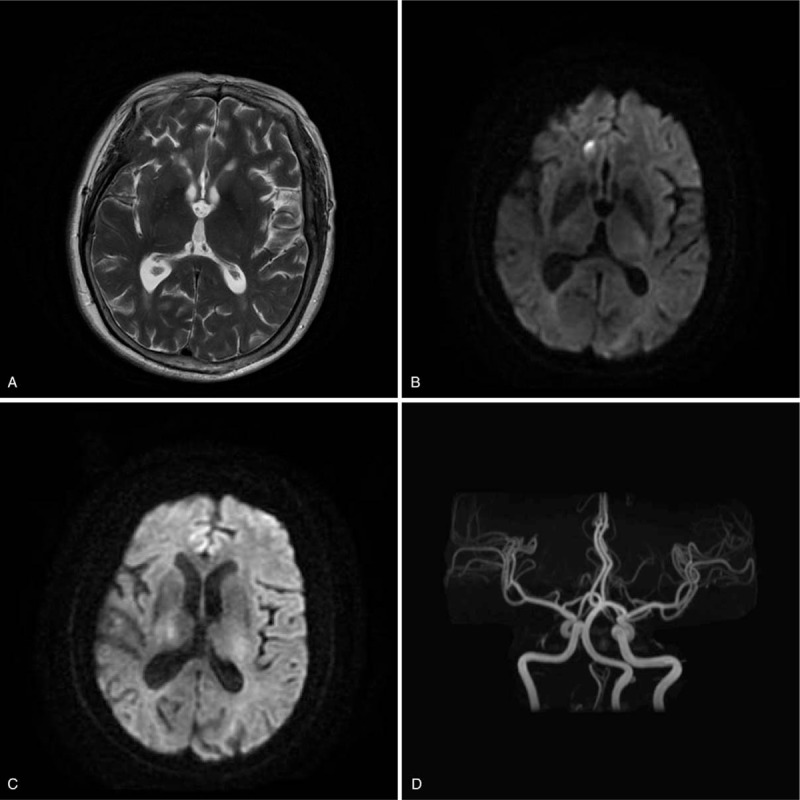
Head MRI performed on September 29th. (A) Patched long T2 signal in the right frontal lobe; (B) high DWI signal in the right frontal lobe; (C) suspicious high DWI signal in bilateral frontal-parietal lobes; (D) Head MRA, suggesting atherosclerotic changes. DWI = diffusion-weighted imaging, MRI = magnetic resonance imaging.

The patient was treated with aspirin, atorvastatin, etimicin, and cefoperazone sulbactam for 3 days, as well as with treatment to improve microcirculation and symptomatic support. By the end of the treatment, her diarrhea was relived, but body temperature still fluctuated between 38.2 and 39.6°C with chills. Neurologically, her state of consciousness deteriorated, her reaction was obviously slower, she was lethargic, her left nasolabial was shallow, flexion was observed when stimulating the extremities and muscle strength was grade 2. A head magnetic resonance imaging (MRI) revealed patched long T1/T2 signal, high diffusion-weighted imaging (DWI) signal, low average diffusion coefficient signal in the right frontal lobe (Fig. 1A and B) and slightly long T1/T2 signal in the bilateral frontal-parietal lobes. Head magnetic resonance angiography (MRA) suggested atherosclerotic changes (Fig. 1D). Neck color Doppler ultrasound showed no abnormality. Thoracic and abdominal CT suggested inflammation in the lower lobe of the lungs. Her stool examination was normal, with weakly positive occult blood.

The patient was then transferred to the Neurosurgical Intensive Care Unit on September 29, 2017 for further diagnosis and treatment. After re-reading her MRI images, suspicious abnormal DWI signals were identified in the bilateral thalamus (Fig. 1C). Lumbar puncture immediately followed, which showed cerebrospinal fluid (CSF) pressure of 180 mmH2O, an elevated protein level of 1486.7 mg/L (normal range, 150–450 mg/L) as well as elevated levels of sugar and chloride. She also had elevated leukocytes at 106 × 10^6^/L (normal range, 0–8 × 10^6^/L), 60.4% monocytes and an electroencephalogram resembling a normal adult at sleep.

She received a series of other tests that confirmed she was positive for anti-hepatitis C virus antibody, and her blood biochemistry, blood coagulation, humoral immunity, and autoantibody tests were normal. She was negative for tuberculosis infecting T cells, blood culture, and cytomegalovirus. Herpes simplex virus immunoglobulin M (IgM) and Brucella antibodies in serum and CSF were negative; however, JEV IgM in blood and CSF both were positive. According to the diagnostic criteria set by Burke et al,^[3,4]^ the patient was diagnosed with JE.

After consulting infectious disease specialists, the patient was treated with human immunoglobulin 0.4 g/kg·d intravenously guttae (ivgtt) for 5 days, as well as with the antiviral drugs acyclovir (500 mg q8h ivgtt) for 3 days and foscarnet sodium (3 g q12h ivgtt) for 13 days. As combined cerebral infarction was also considered, the patient was also treated with platelet aggregation inhibitor Plavix, anti-hyperlipidemic atorvastatin, antibiotics, in addition to reducing body temperature, nutritional support, correction of electrolyte disturbance, and improvement of microcirculation.

**Figure 2:**
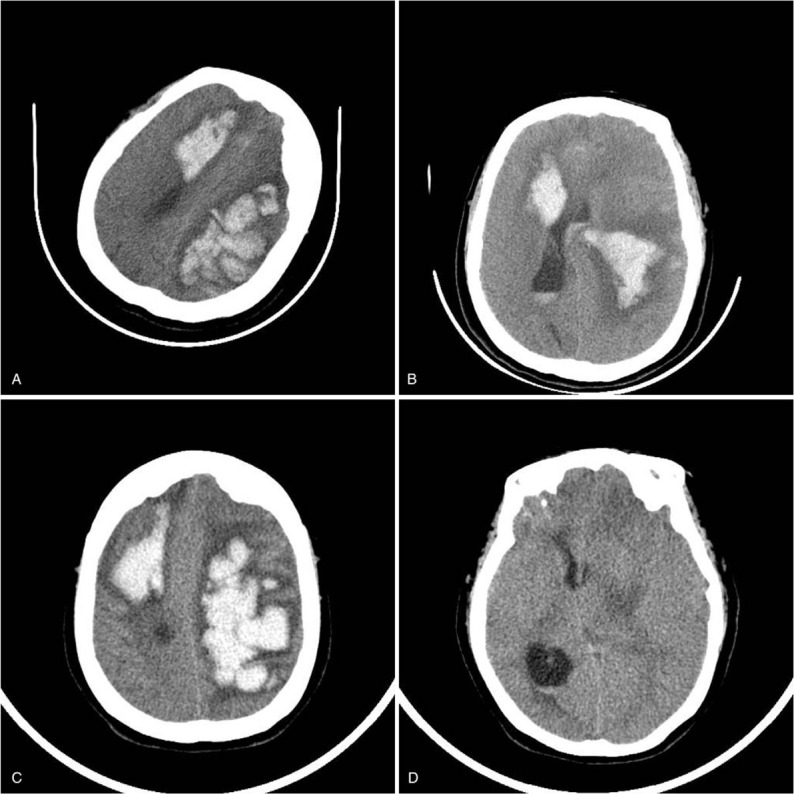
Multiple head CTs. (A) Multiple lobar hemorrhages (October 5th); (B) cerebral hemorrhage of bilateral frontal lobe and left parietal lobe breaking into the ventricle (October 6th); (C) bilateral cerebral hemisphere hemorrhage breaking into the ventricle and subarachnoid space with cerebral hernia (October 12th); (D) slight high density irregular shadow in the right frontal and left parietal lobes and edema in the surrounding brain parenchyma show that the ventricle was obviously under pressure and the brain midline shifted right (October 12th). CT = computed tomography.

On October 1, 2017, the patient's state of consciousness further deteriorated and she went into a coma, along with type II respiratory failure. Tracheal intubation plus ventilator assisted respiration was used. Her body temperature was around 37.0°C. Four days later the patient's systolic pressure rose to around 175 mm Hg with no obvious reason. Neurological examinations suggested no further deterioration. Head CT scan revealed multiple lobar hemorrhages (Fig. 2A). Blood coagulation tests revealed D-dimer was 16.5 mg/L, thrombus map suggested a hypercoagulable state and the inhibition of adenosine diphosphate was 31.4%. Antiplatelet aggregation drugs were discontinued, and the patient received dehydrants of mannitol 125 mL (q6h ivgtt) + torasemide (10 mg q12h intravenous injection (iv), and continuous pump feeding of urapidil that controlled her blood pressure at 160 mm Hg. Surgery was refused by the patient's relatives due to high surgical risk. On October 6th, the patient went into a moderate-deep coma. A CT scan revealed a cerebral hemorrhage breaking into the ventricle (Fig. 2B). The dose of dehydrants was adjusted to mannitol (250 mL q6h ivgtt) + torasemide (10 mg q12h iv). Four days later, considering the renal impairment by mannitol in elderly patients, the dose of dehydrants was further adjusted to mannitol (125 mL q6h ivgtt) for 3 days + torasemide (10 mg q12h iv) for 3 days + glycerol fructose (250 mL q12h ivgtt) for 3 days.

On October 12, 2017, the sizes of the patient's bilateral pupils were uneven, and CT examination suggested cerebral hernia (Fig. 2C and D). The intracranial hematoma was drained immediately, producing 55 mL of bloody fluid, and nutritional neurotherapy and dehydrants for reducing craniofacial pressure discontinued until November 5, 2017.

During hospitalization, the patient had complications that included a multidrug-resistant bacteria infection, liver and kidney dysfunction, electrolyte disturbance, intestinal flora imbalance, hypoproteinemia, and venous thrombosis of the lower extremity, which was actively treated, and a tracheotomy was also performed. When the patient's symptoms had improved, the use of a ventilator was discontinued intermittently, and the longest endurance time was 6 hours. The patient was transferred to another hospital in Beijing on November 7, 2017 at the request of the patient's family members. At the time of transfer, the patient was in a coma and her vital signs were stable. There was spontaneous eye-opening, but the eyeballs could not rotate in response to surrounding sound, and muscle tension of the limbs was weak. Her upper limbs had no response to pain irritation, while the muscles of the lower limbs could contract.

### 2.3 Follow up

The patient was followed with after 1 month, and she had received the second intracranial hematoma drainage through a fine hole due to increased intracranial hemorrhage in the other hospital. The patient was able to tolerate disconnection from the ventilator intermittently; however, during these periods her heart rate and blood pressure increased, and thus, sympathetic storm was suspected.

## 3 Discussion

JE is an acute infectious disease caused by JEV with a main pathology of parenchymal inflammation. Swine are an important source of infection. The transmission route from swine to humans is via arthropods, which is mainly *Culex tritaeniorhynchus* in China. Therefore, JE usually occurs in summer and autumn, especially in the months between July and September, which accounts for more than 90% of cases in the entire year.^[5]^ The sudden onset, severe illness, and rapid development of JE seriously harms the health and life of the patients who contract it. Craniocerebral MRI and CSF examination are of great significance for early diagnosis. The manifestations usually observed in head MRIs of JE patients are low T1WI signal, high T2WI signal and high DWI signal in the thalamus, basal ganglia, substantia nigra, cerebellum, pontine, cerebral cortex, and spinal cord, as well as other parts of the cerebral cortex and spinal cord.^[6]^ Lesions in the bilateral thalamus are highly suggestive of JE; however, basilar artery syndrome and primary central nervous system lymphoma also need to be excluded. There can be elevated protein and leukocyte levels in the CSF of JE patients, while the sugar and chloride levels are normal. JEV IgM appears 4 to 7 days after infection and peaks after 2 weeks. The sensitivity and specificity of the IgM antibody captured in enzyme-linked immunosorbent assays are both over 95%, and diagnosis can be confirmed using a single CSF or serum sample.^[7]^

In this case, the patient had light hemiplegia combined with gastrointestinal reaction at the early onset of the disease. Therefore, cerebral infarction caused by insufficient vascular capacity due to stenosis was initially considered; however, the patient's head MRI suggested frontal lobe lesions associated with right central facial palsy, and the cerebral infarction lesion could not fully explain the symptoms and signs of the patient. Hence there was a large possibility of combined intracranial infection. Considering her high fever, a differential diagnosis needed to be made among JE, toxic dysentery, enterovirus meningitis, listerial encephalitis, and herpes simplex encephalitis. The patient had no signs of septic shock and a normal stool routine, which could exclude toxic dysentery. Enterovirus meningitis patients generally recover quickly, and the symptoms can be relieved in 2 to 3 weeks, but the patient's condition continued to worsen. Additionally, her blood and CSF culture for Listeria were negative. In regards to herpes simplex virus encephalitis, the patient had no temporal lobe lesions observed by head MRI and was negative for CSF antibodies, so there was no evidence to diagnose herpes simplex encephalitis. Hashimoto encephalopathy and autoimmune encephalitis can also cause a similar clinical picture, but thyroid peroxidase antibody and autoimmune encephalitis-related antibodies were negative in our patient. Therefore, due to the results of the MRI imaging and a positive test for JE-specific IgM antibody in CSF and blood, the patient was diagnosed with JE.

A review of the literature suggested that JE complicated with intracerebral hemorrhage can manifest as bilateral thalamic and basal ganglia hemorrhage, which is related to vascular injury caused by inflammation and combined intracranial venous sinus thrombosis.^[8,9]^ However, there has been no previous report of JE with multiple lobar hemorrhages. In this case, although mono-antibody treatment was used for only 9 days, the patient's ventilation and coagulation functions suggested hypercoagulability, which was considered unlikely to be related to antiplatelet aggregation treatment. Although serious JEV infection and secondary inflammatory cell infiltration can cause vascular endothelial cell damage leading to intracranial bleeding, it usually causes bilateral thalamic and basal ganglia hemorrhage,^[9]^ which is different from the features of the case. Rereading the patient's CT and MRI results carefully, we found that her head MRI showed ischemic white matter damage, with multiple lobulated hematoma occurring at the same time, and the second intracranial bleeding presented the same characteristics. According to the above clinical characteristics, we thought the severe intracranial hemorrhage was mainly caused by cerebral amyloid angiopathy, which was validated by referring to the Boston criteria.^[10]^ In addition, the patient's hypercoagulable state raised the possibility of hemorrhages secondary to intracranial venous sinus thrombosis. However, due to the patient's high dependency on the respirator and unstable life signs, she could not tolerate magnetic resonance venography, susceptibility-weighted imaging, or a second MRA examination, which was unfortunate for this case.

Currently, the treatment for JE mainly involves improving the symptoms of fever, convulsion, disturbance of consciousness, and respiratory failure, as well as antiviral treatment, although there is no specific anti-JEV drug. It has been found that gamma globulin can prevent early JEV replication in vivo by stimulating the production of substances that neutralize JEV antigens and harmful substances released by JEV in the brain, reducing demyelination and brain edema, and effectively promoting the recovery of the nervous system.^[11,12]^ As for the amyloid angiopathy with hemorrhage, control of blood pressure can significantly inhibit the hematoma enlargement at the early stage.^[13]^ Neurosurgery can significantly improve the prognosis of patients, especially for those under the age of 75 without ventricular expansion.^[14]^

## 4 Conclusion

When evaluating diseases with acute onset in summer and autumn that are accompanied by fever, gastrointestinal symptoms, dyspnea, and unilateral neurological signs, in addition to considering cerebral infarction combined with digestive and respiratory system infection, patients’ history should be carefully taken to further exclude the possibility of intracranial infections such as JE, and a vertebral puncture CSF examination should be performed, if necessary, to make a definite diagnosis and reduce misdiagnosis. Severe JE in elderly patients develops rapidly. Patients with poor vascular basis (especially cerebral amyloid angiopathy) may suffer from severe intracranial hemorrhage. Doctors should evaluate the patients’ blood vessel status and avoid using drugs that may cause severe bleeding.

## Author contributions

**Conceptualization:** Chao Ren.

**Data curation:** Qiaochan Feng, Shaoxia Yu, Xizhuang Bi, Qi Chen, Hong Liu.

**Formal analysis:** Shaoxia Yu.

**Funding acquisition:** Qi Chen, Hong Liu, Lina Guan.

**Methodology:** Chao Ren, Qiaochan Feng, Xizhuang Bi, Qi Chen, Hong Liu.

**Resources:** Chao Ren, Qiaochan Feng, Shaoxia Yu, Xizhuang Bi, Qi Chen.

**Supervision:** Qi Chen, Jiahui Wang, Xuwen Sun, Lina Guan.

**Writing – original draft:** Qiaochan Feng, Xizhuang Bi.

**Writing – review and editing:** Chao Ren, Jiahui Wang, Xuwen Sun, Hong Liu, Lina Guan.
